# Comparative Analysis of Symbiotic Bacterial Diversity and Sublethal Effects of Nitenpyram Against Two Different Cotton Aphids

**DOI:** 10.3390/biology14121684

**Published:** 2025-11-26

**Authors:** Wenjie Li, Wei Cao, Xuanling Wei, Dongsheng Hu, Kailong Yuan, Renfu Zhang, Yongsheng Yao

**Affiliations:** 1 College of Agriculture, Tarim University, Alar 843300, China; liwenjie010719@163.com (W.L.); m15599950597@163.com (X.W.); 19590129452@163.com (D.H.); 19577938885@163.com (K.Y.); 2 Xinjiang Academy of Agricultural and Reclamation Science, Shihezi 832000, China; caoweinky@163.com; 3 Institute of Plant Protection, Xinjiang Academy of Agricultural Sciences, Urumqi 830091, China

**Keywords:** symbiotic bacterial, *Acyrthosiphon gossypii*, *Aphis gossypii*, nitenpyram, sublethal effect

## Abstract

**Simple Summary:**

*Aphis gossypii* (abbreviated as *Ap. gossypii*) and *Acyrthosiphon gossypii* (abbreviated as *Ac. gossypii*) are significant cotton pests, and their control often relies on insecticides. However, the non-lethal effects of these chemicals, particularly on pests and their internal symbiotic bacteria, remain incompletely understood. Our research investigated the impact of sublethal doses of nitenpyram on both aphid species. The results demonstrated a higher toxicity of nitenpyram to *Ac. gossypii*. Exposure negatively affected the survival and reproduction of the immediate generation in both species. Notably, these effects persisted in their offspring but in contrasting ways population growth potential was enhanced in *Ap. gossypii* but suppressed in *Ac. gossypii*. By the third generation, the populations of both species stabilized. At a deeper level, insecticide stress significantly disrupted the symbiotic bacterial communities in both aphids, resulting in consistent changes in the abundance of *Sphingomonas* and *Buchnera* over multiple generations. This study links insecticide-induced shifts in pest population dynamics to alterations in their symbiotic bacteria, offering a novel perspective for managing pest resurgence and resistance.

**Abstract:**

Symbiotic bacteria in insects are known to play crucial roles in detoxification metabolism and adaptation to host plant secondary metabolites. In the cotton-growing region of Xinjiang, China, the *Ap. gossypii* and the *Ac. gossypii* exhibit significant differences in sensitivity or resistance to pesticides. However, whether their detoxification-related symbiotic bacteria change under insecticide stress remains unclear. This study assessed the toxicity of nitenpyram to both aphid species and the effects of LC_20_ treatment on their growth, development, and reproduction. Bacterial community dynamics across generations (G0–G2) were analyzed by 16S rRNA gene amplicon sequencing. The LC_20_ of nitenpyram reduced the longevity and fecundity of the parent generation in both species. In *Ap. gossypii*, the intrinsic rate of increase (*r_m_*), net reproductive rate (*R*_0_), and finite rate of increase (*λ*) increased in the G1–G2 generations, whereas these parameters significantly decreased in *Ac. gossypii*. By the G3 generation, biological parameters in both species showed no significant differences compared to the control. Nitenpyram disrupted the stability of symbiotic bacterial communities in both aphids. In *Ac. gossypii*, *Sphingomonas*, a genus with detoxification potential, was consistently suppressed in G1–G2, while the abundance of the primary symbiont *Buchnera* initially decreased sharply and subsequently recovered. In contrast, the bacterial community in *Ap. gossypii* remained largely stable. These findings indicate that sublethal concentrations of nitenpyram exert distinct transgenerational effects on the two aphid species and disrupt the stability of their symbiotic bacterial communities.

## 1. Introduction

*Aphis gossypii* and *Acyrthosiphon gossypii* are major piercing-sucking pests of cotton, classified as species with similar ecological niches and seasonal activity patterns in cotton fields, with their populations exhibiting temporal differences [1,2,3]. Both adults and nymphs use their slender stylets to extract phloem sap from host plants, primarily affecting crops in the Malvaceae, Fabaceae, Brassicaceae, and Asteraceae families [4]. Early studies indicated that *Ac. gossypii* primarily infests cotton from the seedling to squaring stages, whereas *Ap. gossypii* becomes the dominant species during the bud-to-flowering period, although it occurs throughout the entire cotton growth cycle [5]. In recent years, *Ap. gossypii* has dominated throughout the entire cotton growth cycle [6]. Chemical control remains a critical strategy for managing aphid populations due to their rapid population growth.

Nitenpyram, a novel neonicotinoid insecticide, disrupts nerve signal transduction by binding to the insect’s nicotinic acetylcholine receptors (nAChRs) [7,8]. Developed and commercialized by Takeda Chemical Industries in 1995, nitenpyram has been widely used in China to control aphid [9,10]. Its high efficacy against piercing-sucking pests has established it as an essential tool in aphid management [11,12]. Different levels of resistance to nitenpyram have been reported in field pest populations [13,14]. This resistance is not restricted to aphids. In several Asian regions, the brown planthopper (*Nilaparvata lugens*) has developed elevated resistance following prolonged and intensive nitenpyram application [15]. Similarly, certain populations of the Middle East–Asia Minor 1 (MEAM1) cryptic species of *Bemisia tabaci* have exhibited moderate to high resistance to nitenpyram [16]. Additionally, globally significant pests such as *Myzus persicae* have developed resistant populations in multiple regions, with resistance mechanisms frequently associated with enhanced detoxification enzyme activity [17]. As nitenpyram degrades in the environment and resistance evolves, exposure of aphids to sublethal concentrations of nitenpyram may increase the risk of resistance evolution.

With the degradation of insecticides and the continuous evolution of insect resistance, the toxicity of insecticides in the environment may decline to sublethal concentrations over time, thereby producing sublethal effects on surviving individuals. These effects could potentially influence the symbiotic relationships between insects and their associated microorganisms under insecticide-induced stress [18]. On one hand, sublethal effects generally negatively impact pest life table parameters. For example, cotton aphid fecundity was reduced to varying degrees by exposure to nitenpyram, dinotefuran, clothianidin, thiacloprid, thiamethoxam, and acetamiprid [19]. Additionally, sublethal concentrations of flonicamid significantly decreased the intrinsic rate of increase (*r_m_*) and finite rate of increase (*λ*) of *Ap. gossypii* populations compared to controls, effectively suppressing population growth [20]. On the other hand, sublethal concentrations can induce hormesis, increasing the reproductive output and longevity of surviving individuals [21]. Hormesis is considered a critical factor in pest resurgence. Studies have demonstrated that sublethal concentrations of acetamiprid, thiamethoxam, imidacloprid, and nitenpyram stimulate reproduction in *Ap. gossypii* [22,23,24,25].

Insecticides directly affect pests, often producing transgenerational effects that significantly influence symbiotic bacteria. Consequently, insect resistance interacts closely with symbiotic bacteria, which may assist pests in metabolizing toxins and enhancing their tolerance to insecticides. Symbiont-mediated pesticide detoxification mechanisms have been documented across various insect taxa, with symbiotic bacteria contributing to the development of insecticide resistance [26,27]. For instance, *Sphingomonas paucimobilis* abundance is higher in imidacloprid-resistant *Ap. gossypii* populations compared to susceptible ones, facilitating resistance evolution [28]. The plasmid encoding SaxA in the gut microbiome of *Delia radicum* and the *Bactrocera dorsalis* gut symbiont *Citrobacter* sp. have been shown to aid in chemical detoxification by regulating the expression of genes encoding detoxification enzymes [29,30]. Additionally, *Corynebacterium* sp. 2-TD in *Helicoverpa armigera* can directly metabolize exogenous toxins [31]. A close and complex symbiotic relationship exists between aphids and their endosymbiotic bacterial communities, which plays a crucial role in host adaptive evolution. In addition to primary symbionts (*Buchnera*) that provide essential nutrients, various secondary symbionts have been demonstrated to influence aphid resistance to parasitoids and thermal stress [32,33]. Increasing evidence suggests that insecticide stress, as a strong selective pressure, can alter the symbiotic bacterial community structure in aphids, and the enrichment of specific symbionts may be directly associated with enhanced host detoxification capacity [34]. Consequently, examining changes in the symbiotic bacterial community under insecticide stress is critical for understanding the toxicological mechanisms underlying aphid resistance development.

As an effective tool for studying the sublethal effects of insecticides on pest populations [35], life tables accurately reflect the population dynamics of insects across various developmental stages and their entire life cycle by integrating and analyzing biological parameters such as life expectancy, mortality, and fecundity [36,37]. This study evaluated the effects of LC_20_ nitenpyram on the biological traits and bacterial communities of *Ap. gossypii* and *Ac. gossypii*. By exposing parental aphids to sublethal concentrations of nitenpyram, the sublethal effects on biological characteristics and population parameters across three successive generations were investigated. Furthermore, to elucidate the underlying mechanisms of the differential sensitivity to nitenpyram and its transgenerational effects between *Ap. gossypii* and *Ac. gossypii* at the microbial level, we employed 16S rRNA gene sequencing to assess the impact of the insecticide on the structure and composition of their symbiotic bacterial communities. The findings of this research enhance our understanding of changes in interspecific relationships between *Ap. gossypii* and *Ac. gossypii* under insecticide stress, providing theoretical insights into the molecular mechanisms underlying aphid adaptation to insecticides.

## 2. Materials and Methods

### 2.1. Insect Rearing and Insecticide

*Aphis gossypii* and *Acyrthosiphon gossypii* were collected from the agricultural experimental field of Tarim University, located at the eastern gate of Alar City, Xinjiang. To establish standardized experimental populations, healthy aphids from the collection were individually transferred to potted cotton plants (Xinluzhong 67) confined within mesh cages (60 × 60 × 60 cm) and maintained in a climate-controlled chamber (BIC-400) for more than five consecutive generations. Aphids of uniform size and health from these cultures were subsequently selected for bioassays. The environmental conditions were set at temperature (22 ± 1) °C, relative humidity (70 ± 5) %, and a photoperiod of 16:8 (L:D) To ensure optimal nutrition and avoid crowding stress, fresh, insect-free potted cotton plants were provided every 7–10 days. All tests used insects of consistent physiological status.

The test compounds consisted of 95% technical-grade nitenpyram obtained from Shandong Hailir Chemical Co., Ltd., (Weifang, China) and Triton X-100 surfactant, which was acquired from Beijing Solarbio Science & Technology Co., Ltd. (Beijing, China)

### 2.2. Bioassays

The toxicity of nitenpyram against *Ap. gossypii* and *Ac. gossypii* was determined using the leaf-dipping method [38]. Acetone was used to dissolve the technical-grade nitenpyram, followed by sequential dilution with distilled water containing 0.1% Triton X-100 to prepare six treatment concentrations. The specific concentrations tested for *Ap. gossypii* were 35 mg L^−1^, 17.5 mg L^−1^, 8.75 mg L^−1^, 4.38 mg L^−1^, 2.19 mg L^−1^, 1.09 mg L^−1^, and for *Ac. gossypii* were 70 mg L^−1^, 35 mg L^−1^, 17.5 mg L^−1^, 8.75 mg L^−1^, 4.38 mg L^−1^, 2.19 mg L^−1^. These concentrations were selected based on preliminary range-finding tests to ensure they adequately covered the dose-response curve, from minimal to high mortality, and were prepared via a serial dilution method. Fully expanded, tender cotton leaves were harvested from the 4th to 5th nodal position of cotton plants at the budding stage. Leaf discs (9 cm) were punched out from these leaves. The leaf discs were immersed in the respective test solutions for 15 s, air-dried, and placed face-down in Petri dishes (9 cm diameter × 1.5 cm height) containing a solidified 1.8% agar layer. The agar was used to maintain leaf turgor and freshness throughout the 48-h bioassay period, a technique adapted from [38]. Thirty aphids of each species, selected for uniform age, size, vigor, and wingless morphology were introduced into each dish. Ref. [38], To maintain a consistent aphid population, the dish openings were covered with paper towels. The experiment was conducted in an artificial climate chamber under conditions identical to those used for rearing. The control treatment consisted of distilled water with 0.1% Triton X-100. Each treatment included three biological replicates, and aphid mortality was recorded after 48 h. The LC_20_, LC_50_, and corresponding 95% confidence intervals were calculated.

### 2.3. Sublethal Effects of Nitenpyram on Aphis gossypii and Acyrthosiphon gossypii

A minimum of 300 aphids were placed in an artificial climatic chamber, and the newly produced aphids after 24 h were designated as the G0 generation to ensure a consistent age stage. These aphids were reared to adulthood to serve as the insect source for determining the sublethal effects of nitenpyram. Based on the results of the virulence assay, fresh cotton leaves were treated with nitenpyram at the LC_20_ concentration (sublethal dose) for 15 s. After drying, a corresponding number of wingless aphids were transferred into Petri dishes and placed in an incubator. Surviving individuals were moved to fresh, untreated leaves after 48 h. One aphid was placed in each dish, with a minimum of 30 neonatal aphids (<24 h) per group. The control group consisted of distilled water containing 0.1% Triton X-100. To facilitate efficient recording of survival and reproduction, each replicate under each treatment was assigned a unique identifier. Cotton leaves and Petri dishes were replaced every 3 days throughout the experiment. The study began with the exposure of two parental aphids (G0) to nitenpyram and continued until the completion of the G3 generation.

### 2.4. Determination of Symbiotic Bacteria in Aphis gossypii and Acyrthosiphon gossypii Using Nitenpyram

Wingless adults from both the aphid control and treatment groups of the G0–G2 generations were collected into 1.5 mL freezing tubes for 16S rRNA sequencing. Each treatment was replicated three times. To minimize in vitro microbial effects on aphids, each aphid was rinsed with 75% ethanol for 1 min, followed by three washes with sterile water. Post-treatment, genomic DNA from *Ap. gossypii* and *Ac. gossypii* was extracted using the MagPure Soil DNA LQ Kit (Magen Biotechnology Co., Ltd., Guangzhou, China; D6356-02). The concentration and quality of DNA from both aphid species were assessed using 1% agarose gel electrophoresis and a NanoDrop 2000 (Thermo Fisher Scientific Inc., Waltham, MA, USA) spectrophotometer, respectively.

PCR amplification of *Ap. gossypii* and *Ac. gossypii* was conducted using universal primers targeting the 16S rRNA V3–V4 region (343F: TACGGRAGGCAGCAG; 798R: AGGGTATCTAATCCT) [39]. The first and second rounds of PCR amplification, including reaction systems, procedures, electrophoretic detection, purification, and quantification of PCR products, were performed as previously described [28]. High-throughput sequencing was carried out by Shanghai Ouyi Biomedical Technology Co. The raw sequencing data for *Ap. gossypii* and *Ac. gossypii* were deposited in the GenBank SRA database in FASTQ format.

### 2.5. Statistical Analysis

The bioassay results of nitenpyram on *Ap. gossypii* and *Ac. gossypii* were analyzed using IBM SPSS Statistics 26.0 software, and LC_20_ and LC_50_ values (95% confidence intervals) were calculated. Growth, development, and reproduction data were analyzed using TWOSEX-MSChart software (Version 2019) [40]. Means and standard errors of life table parameters were estimated after 100,000 random resampling (bootstrap procedure), and significant differences between aphid control and treatment groups were analyzed using a paired bootstrap test procedure [41,42,43]. Survival, fecundity, and life expectancy curves of *Ap. gossypii* and *Ac. gossypii* were plotted using SigmaPlot 14.0 based on life table data.

Raw data sequences of *Ap. gossypii* and *Ac. gossypii* were trimmed, quality-filtered, noise-reduced, spliced, and de-chimerized. The obtained representative sequences and ASV abundance tables were compared and annotated in the SILVA database (version 138). Species diversity among bioenvironmental samples and between different sample subgroups was assessed using alpha and beta diversity analyses [44]. Correlations between the two aphid control and treatment groups were evaluated using principal coordinate analysis (PCoA) [45]. LEfSe analysis (LDA) was employed to identify differences in species composition between groups [46]. Additionally, species differences were analyzed using SPSS 26.0 software at the *p* < 0.05 significance level.

## 3. Results

### 3.1. Toxicity of Nitenpyram to Aphis gossypii and Acyrthosiphon gossypii

The LC_50_ of nitenpyram for *Ac*. *gossypii* was 10.12 mg L^−1^, which was significantly lower than the 28.62 mg L^−1^ required for *Ap*. *gossypii*. This result indicates that *Ap. gossypii* exhibited 2.83-fold greater tolerance to the insecticide than *Ac. gossypii*. This trend was even more pronounced at the LC_20_ level, where the concentration for *Ap. gossypii* (8.73 mg L^−1^) was 3.51 times higher than that for *Ac. gossypii* (2.49 mg L^−1^), demonstrating the greater efficacy of nitenpyram against *Ac. gossypii* across concentrations (Table 1). In this study, the LC_20_ of nitenpyram was used to evaluate the statistical traits of *Ap*. *gossypii* and *Ac*. *gossypii* as well as the effects of symbiotic bacteria.

### 3.2. Sublethal Effects of Nitenpyram on Fecundity and Longevity of Aphis gossypii and Acyrthosiphon gossypii Parents (G0)

**Figure 1:**
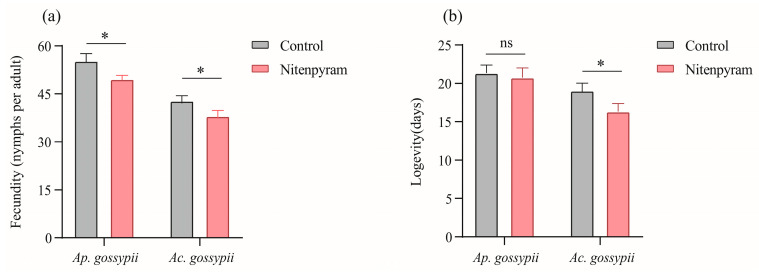
Fecundity (**a**) and longevity (**b**) of *Aphis gossypii* and *Acyrthosiphon gossypii* G0 after treatment with nitenpyram. * Significant difference (*p* < 0.05), ns, no significant difference.

The fecundity and longevity of *Ap*. *gossypii* and *Ac*. *gossypii* parents were assessed after 48 h of treatment with nitenpyram LC_20_ (Figure 1). The results demonstrated that nitenpyram significantly reduced the fecundity and longevity of *Ac. gossypii* compared to the control. Additionally, the individual fecundity of the *Ap. gossypii* treated group was significantly lower than that of the control, whereas longevity was not significantly affected.

### 3.3. Sublethal Effects of Nitenpyram on Aphis gossypii and Acyrthosiphon gossypii, G1–G3 Generation

Exposure of *Ap. gossypii* and *Ac*. *gossypii* parents to nitenpyram affected the growth, development, and reproduction of the progeny (G1–G2) generations of both aphids (Table 2). The adult pre-oviposition period (APOP), total pre-oviposition period (TPOP), and pre-adult stages of *Ap. gossypii* progeny in the G1 and G2 generations were significantly shorter after treatment with nitenpyram. Compared with the control group, nitenpyram prolonged the TPOP and significantly reduced the APOP of individuals in the G1–G2 generations of *Ac. gossypii*. Additionally, the fecundity of *Ap. gossypii* was significantly increased by the treatment compared to the control, whereas the fecundity of *Ac. gossypii* individuals was significantly reduced. Furthermore, nitenpyram decreased the longevity of the G1 and G2 generations of both aphids. Compared to the control, nitenpyram treatment significantly increased the intrinsic rate of increase (*r_m_*), finite rate of increase (*λ*), and net reproductive rate (*R*_0_) of *Ap. gossypii* G1 and G2 generation populations, whereas *r_m_*, *λ*, and *R*_0_ of *Ac. gossypii* were significantly reduced. The mean generation time (*T*) of *Ap. gossypii* decreased in the G1 and G2 generations after treatment, whereas *Ac. gossypii* (*T*) increased compared to the control. By the G3 generation, the sublethal effect of the treatment diminished, and no significant differences were observed in the biological characteristics of the G3 generation populations of either aphid compared to the control (Table 3).

**Figure 2:**
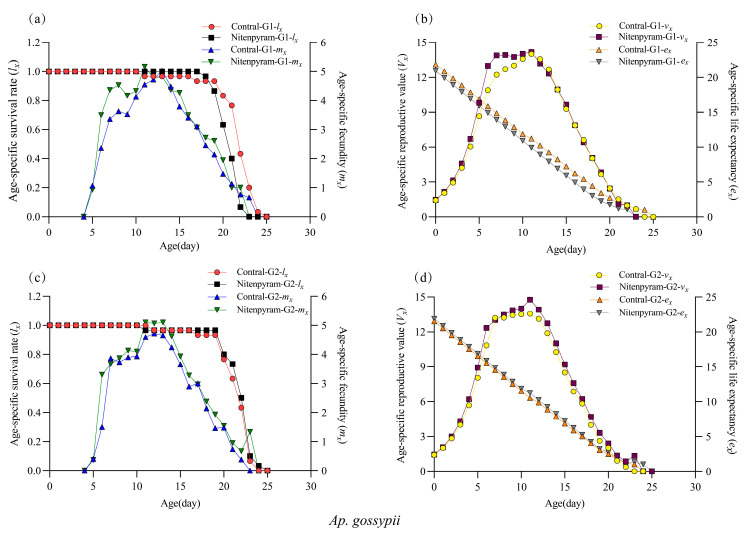
Age-specific survival rate (*l_x_*), Age-specific fecundity (*m_x_*), Age-specific reproductive value (*V_x_*) and Age-specific life expectancy (*e_x_*) of *Aphis gossypii* in G1 and G2 generation after 48 h exposure. (**a**) Age-specific survival rate (*l_x_*) and Age-specific fecundity (*m_x_*) of G1 generation; (**b**) Age-specific reproductive value (*V_x_*) and Age-specific life expectancy (*e_x_*) of G1 generation; (**c**) Age-specific survival rate (*l_x_*) and Age-specific fecundity (*m_x_*) of G2 generation; (**d**) Age-specific reproductive value (*V_x_*) and Age-specific life expectancy (*e_x_*) of G2 generation.

**Figure 3:**
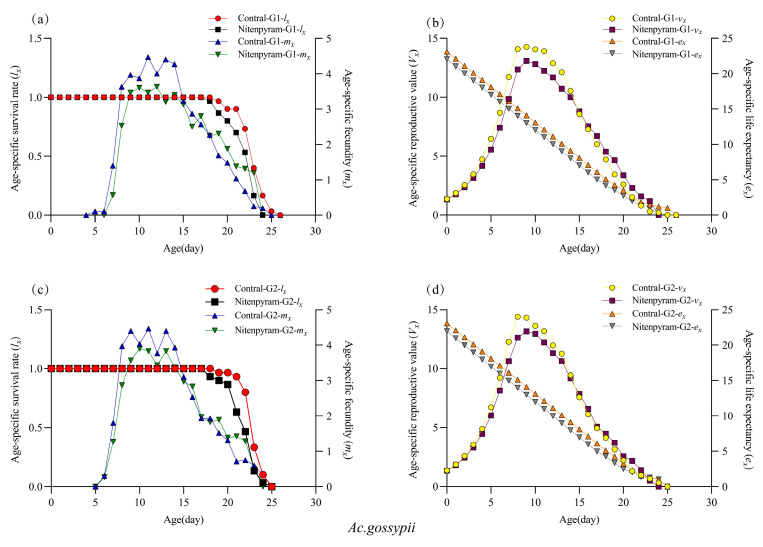
Age-specific survival rate (*lx*), Age-specific fecundity (*mx*), Age-specific reproductive value (*Vx*) and Age-specific life expectancy (ex) of *Acyrthosiphon gossypii* in G1 and G2 generation after 48 h exposure. (**a**) Age-specific survival rate (*l_x_*) and Age-specific fecundity (*m_x_*) of G1 generation; (**b**) Age-specific reproductive value (*V_x_*) and Age-specific life expectancy (*e_x_*) of G1 generation; (**c**) Age-specific survival rate (*l_x_*) and Age-specific fecundity (*m_x_*) of G2 generation; (**d**) Age-specific reproductive value (*V_x_*) and Age-specific life expectancy (*e_x_*) of G2 generation.

Age-specific survival (*l_x_*) curves for the two aphid treatment groups and the control are presented in Figure 2a,c. The *l_x_* curves for the G1 and G2 generations of *Ap. gossypii* and *Ac. gossypii* exhibited a decline with increasing age (*X*). The *l_x_* curves of the G1 generation of *Ap. gossypii* demonstrated a delayed decline in the parental treatment with nitenpyram LC_20_ compared to the control, while the survival rates of the G2 generation individuals showed significant overlap. The age-specific survival of *Ac. gossypii* G1–G2 generations decreased significantly by day 18 relative to the control (Figure 3a,c). The age-specific fecundity (*m_x_*) of both aphid species displayed an initial increase followed by a decrease with advancing age. The *m_x_* curves of the *Ap. gossypii* G1–G2 generations peaked at day 11 (5.17) and day 11 (5.10), respectively, following nitenpyram treatment, which were higher than those of the control group at day 13 (5.03) and day 12 (4.72) (Figure 2a,c). The maximum *m_x_* values for the *Ac. gossypii* G1–G2 generations were observed at day 12 (3.63) and day 10 (3.90), respectively, after nitenpyram exposure, occurring later than those of the control (Figure 3a,c).

### 3.4. Diversity of Symbiotic Bacteria of Aphis gossypii and Acyrthosiphon gossypii Under Nitenpyram Exposure

**Figure 4:**
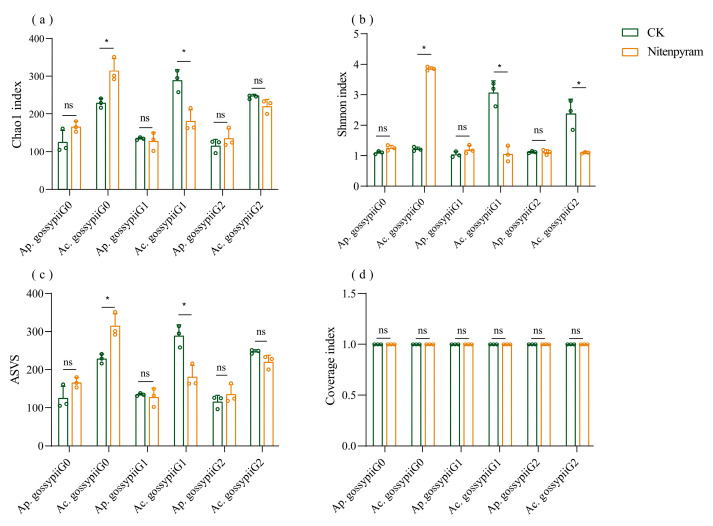
Differences in alpha diversity of bacterial communities between *Aphis gossypii* and *Acyrthosiphon gossypii*. * p < 0.05; ns: not significant. (**a**–**d**) represent the alpha diversity of the bacterial communities in Chao 1, Shannon, ASVS and Goods_coverage, respectively. The bar diagram is generated according to the data in Table S1.

The V3–V4 hypervariable regions of the 16S rRNA gene amplicons from *Ap. gossypii* and *Ac. gossypii* G0–G2 generations were sequenced using the Illumina platform. A total of 465,796 and 462,365 quality-filtered reads were obtained from all *Ap. gossypii* and *Ac. gossypii* samples, respectively. The Goods_coverage values for both aphid species reached 1, indicating sufficient sequencing depth. Following nitenpyram treatment, *Ac. gossypii* exhibited significant changes in symbiotic bacterial ASV abundance across its G0 and G1 generations, whereas *Ap. gossypii* showed no significant changes over three consecutive generations. Alpha diversity analysis was used to compare species diversity between the aphicide-treated groups and the control group. In *Ac. gossypii*, the Chao 1 and Shannon indices increased significantly in the G0 generation after nitenpyram exposure but decreased significantly in the G1 generation. The Shannon indices further declined significantly in the G2 generation. In contrast, the bacterial community composition of *Ap. gossypii* remained stable across three consecutive generations (Figure 4, Table S1).

### 3.5. Microbial Community of Aphis gossypii and Acyrthosiphon gossypii Under Nitenpyram Exposure

**Figure 5:**
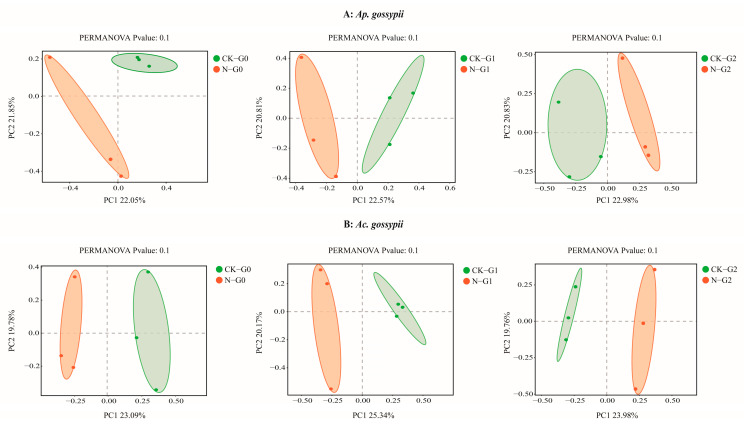
Principal coordinates analysis (PCoA) of *Aphis gossypii* and *Acyrthosiphon gossypii* G0–G2 bacteria based on Bray-Curtis distance. The green and orange circles represent the control group and the treatment group.

Principal coordinates analysis (PCoA) revealed that the effects of nitenpyram on microbial community structure differed between the two aphid species and across treatment concentrations (Figure 5). The distance between control and treated groups was significantly greater for *Ac. gossypii* than for *Ap. gossypii* across three consecutive generations following nitenpyram treatment. As the transgenerational effects diminished, the disparity between control and treated groups decreased, with the distance between groups becoming smaller in the *Ap. gossypii* G2 generation. These findings suggest that the symbiotic bacterial community structures of both aphid species return to a stable state within a defined time period.

The composition of symbiotic bacterial communities changed across three consecutive generations in both aphid species following nitenpyram exposure (Tables S2–S4). The bacterial communities of *Ap. gossypii* and *Ac. gossypii* were primarily distributed among three phyla: Proteobacteria, Bacteroidetes, and Firmicutes, with Proteobacteria being the dominant phylum in both species. In *Ac. gossypii*, Proteobacteria abundance exhibited a pattern of initial decrease followed by increase after nitenpyram treatment, whereas no significant change was observed in *Ap. gossypii*.

**Figure 6:**
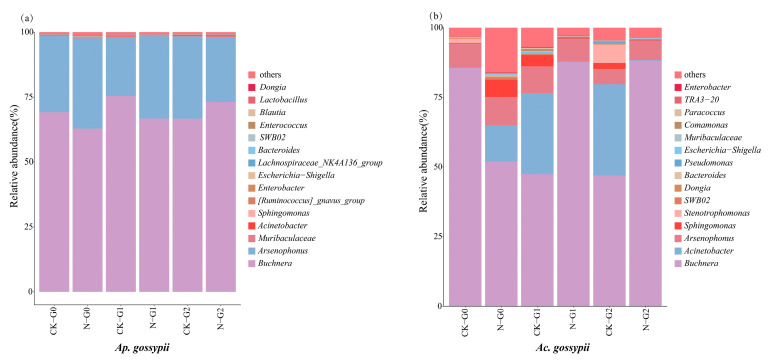
Relative abundance barplot of the top 15 bacterial communities in three successive generations at genus levels of *Aphis gossypii* and *Acyrthosiphon gossypii*. (**a**,**b**) show the histograms of *Aphis gossypii* and *Acyrthosiphon gossypii* species abundance at the levels of genus, respectively. The barplot is generated according to the data in Tables S5–S7.

The Barplot illustrates the composition and abundance of the microbial community in all samples of both aphid species at the genus level. Among the top 15 most abundant bacterial genera, *Ap. gossypii* and *Ac. gossypii* shared 10 common genera while each species possessed 5 unique genera (Figure 6, Tables S5–S7). As the primary symbiont of aphids, *Buchnera* dominated the microbial communities of both aphid species. Following exposure to nitenpyram (LC_20_), the abundance of *Buchnera* in *Ac. gossypii* significantly decreased in the G_0_ generation (*p* < 0.001) but rebounded in G1 and G2 generations (*p* < 0.05), whereas no significant changes were observed in *Ap. gossypii* across all generations (*p* > 0.05).

Concurrently, *Sphingomonas* exhibited a significant decline in *Ac. gossypii* during G1 (*p* < 0.05), while no significant alteration was detected in *Ap. gossypii* (*p* > 0.01). For *Acinetobacter*, a significant increase was observed in *Ac. gossypii* at G_0_ (*p* < 0.01), followed by significant decreases in G1 and G2 (*p* < 0.05). In contrast, *Ap. gossypii* only showed a significant reduction in G1 (*p* < 0.01), with non-significant increases in other generations (*p* > 0.05). Both aphid species demonstrated significant reductions in Muribaculaceae abundance during G1 (*p* < 0.05). By G2, while both treated groups showed increased Muribaculaceae relative abundance compared to controls, the difference remained non-significant in *Ac. gossypii* (*p* > 0.05).

**Figure 7:**
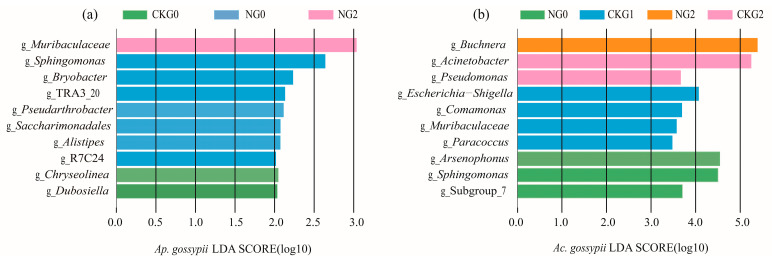
Identification of differentially abundant bacteria between the control group and the nitenpyram group in *Aphis gossypii* and *Acyrthosiphon gossypii*. (**a**,**b**) were abundant species in three successive generations of *Aphis gossypii* and *Acyrthosiphon gossypii*, respectively. LDA, linear discriminant analysis.

Biomarkers of *Ap. gossypii* and *Ac. gossypii* were analyzed using LEfSe (LDA score > 2.0) to reveal changes in dominant populations within their microbial communities under nitenpyram stress and to identify taxa associated with nitenpyram (Figure 7). The dominant and inhibitory populations of *Ap. gossypii* and *Ac. gossypii* were identified at the genus level following nitenpyram treatment.

In *Ap. gossypii*, *Chryseolinea* and *Dubosiella* were identified in the control G0 generation. *Sphingomonas*, Saccharimonadales, *Bryobacter*, *Alistipes*, *Pseudarthrobacter*, TRA3_20, and R7C24 were significantly enriched in the G0 generation of the treatment group after nitenpyram exposure. The abundance of Muribaculaceae in the G2 generation of the treatment group increased significantly. In *Ac. gossypii*, *Arsenophonus* and *Sphingomonas* were identified in the G0 generation after nitenpyram exposure. A significant increase in the abundance of *Escherichia*-*Shigella*, *Comamonas*, Muribaculaceae, and *Paracoccus* was observed in the G1 generation control. *Acinetobacter* and *Pseudomonas* were enriched in the G2 generation controls. *Buchnera* was identified in the *Ac. gossypii* G2 generation treatment group.

## 4. Discussion

In this study, we investigated the cross-generational sublethal effects of sublethal concentrations of nitenpyram on *Ap. gossypii* and *Ac. gossypii*. The bioassay results demonstrated that the 48-h toxicity values (LC_20_) for the two aphid species were 8.73 mg L^−1^ and 2.49 mg L^−1^, respectively, while the LC_50_ values were 28.62 mg L^−1^ and 10.12 mg L^−1^, respectively. Using the LC_20_ value as the sublethal concentration, nitenpyram exhibited greater toxicity to adult *Ac. gossypii* compared to *Ap. gossypii*. The observed differences in toxicity values between the two aphid species may primarily be attributed to species-specific variations, individual differences, and differential sensitivity to insecticides.

Exposure to insecticides tends to negatively affect the individual biology of most pests, in addition to direct lethal effects. The results of the life history parameter assessments revealed that the longevity and fecundity of *Ac. gossypii* (G0 generation) adults were significantly reduced after 48 h of treatment with nitenpyram (LC_20_), and a similar trend was observed in the longevity and fecundity of *Ap. gossypii* (G0 generation) adults. This may be attributed to aphids reducing fecundity and enhancing individual survival as an adaptive response to nitenpyram-induced stress. Similarly, direct exposure of *Ap. gossypii* to afidopyropen [47], nitenpyram [24], and thiamethoxam [25] also reduced adult longevity and fecundity. These findings suggest that nitenpyram exerts an inhibitory effect on the longevity and fecundity of both *Ap. gossypii* and *Ac. gossypii* (G0 generation).

Nitenpyram significantly influenced the biological characteristics of *Ap. gossypii* and *Ac. gossypii* across G1–G2 generations. *Ac. gossypii* exposed to nitenpyram exhibited prolonged developmental durations in both G1 and G2 generations at all life stages. A comparable phenomenon was observed following imidacloprid treatment in wheat aphids [48]. Concurrently, population parameters including the *r_m_*, *λ*, and *R*_0_ were significantly reduced, while the *T* increased markedly. Exposure to sublethal concentrations of imidacloprid also decreased the *r_m_* and λ in *Rhopalosiphum padi* [49] and *Myzus persicae* [50], thereby suppressing population growth. Similarly, treatments with afidopyropen [38] and flonicamid [51] reduced the growth traits of *Ap. gossypii*. These findings suggest that insects reallocate energy toward detoxifying chemical insecticides at the expense of normal developmental processes [52,53].

However, *Ap. gossypii* exhibited differential responses across various life table parameters. When parental aphids (G0) were exposed to sublethal concentrations of nitenpyram (LC_20_), the APOP, TPOP, and *T* of *Ap. gossypii* G1 and G2 were reduced, while the *R*_0_, *λ*, and *r_m_* of progeny (G1–G2) were significantly elevated. Previous studies have demonstrated that exposure to sulfoxaflor similarly increased *R*_0_ and fecundity in *Ap. gossypii* [54]. Comparable findings were reported following acetamiprid treatment of soybean aphids [55]. These effects may arise from the disruption of insect physiological homeostasis after parental exposure to insecticides [24], wherein fecundity increases due to overcompensatory responses in the G1 generation, sublethal effects diminish in G2, and populations of *Ap. gossypii* and *Ac. gossypii* stabilize by G3. Our findings indicate that low-dose pesticide exposure may enhance biological adaptation in insects. Such biphasic effects induced by sublethal insecticide concentrations reflect evolutionary adaptations under environmental stress, enabling insects to better tolerate subsequent exposure to higher insecticide concentrations. This phenomenon may facilitate the resurgence or reinfestation of pest populations [56].

Insect symbiotic bacteria play significant physiological roles in their hosts [57,58]. In this study, the bacterial communities of *Ap. gossypii* and *Ac. gossypii* across three consecutive generations (G0–G2) were found to be primarily distributed among three phyla—Proteobacteria, Bacteroidetes, and Firmicutes—following parental exposure to a sublethal concentration of nitenpyram (LC_20_). Proteobacteria emerged as the dominant phylum. Previous studies have reported that Proteobacteria is the most abundant phylum in insect bacterial communities [59] and contributes to maintaining normal insect growth and development [60]. Sequencing data revealed a decline in symbiotic bacterial abundance across three consecutive generations (G0–G2) of *Ap. gossypi* under nitenpyram stress, whereas *Ac. gossypii* exhibited an initial increase followed by a decrease (Tables S2–S4). The trends observed in our three-generation experiment align with findings from longer-term studies. For example, a study on parasitoid wasps demonstrated that exposure to sublethal pesticide levels over 36 generations led to heritable shifts in the gut microbiota, which in turn conferred herbicide resistance to their offspring [61]. Similarly, research on the brown planthopper (*Nilaparvata lugens*) revealed that six consecutive generations of exposure to the LC_20_ of nitenpyram not only enhanced insecticide tolerance and significantly increased fecundity but also suggested profound physiological adaptations, potentially including alterations in symbiotic relationships [62]. Furthermore, principal coordinates analysis (PCoA) demonstrated significant separation between the treatment groups of both *Ap. gossypii* and *Ac. gossypii* and their respective control groups, indicating that nitenpyram disrupted the stability of the symbiotic bacterial communities in these aphid species.

Our results demonstrate that nitenpyram stress significantly altered the abundance of key symbiotic bacteria in *Ap. gossypii* and *Ac. gossypii*, potentially affecting host physiological homeostasis and adaptation. Notably, *Buchnera*—the primary nutritional symbiont—exhibited species-specific responses: its abundance was significantly suppressed in *Ac. gossypii* at G0 but increased above control levels in G1–G2, whereas it remained stable across all three generations in *Ap. gossypii*. The sharp decline in *Buchnera* abundance in *Ac. gossypii* at G0 likely disrupted its role in supplying essential nutrients. Previous studies have demonstrated that chemical stressors such as avermectin [63], deltamethrin [64], and trifloxystrobin [65] alter *Buchnera* abundance, and its reduction impairs the synthesis of vitamins and essential amino acids [59,66,67], increasing host susceptibility to insecticides [63]. Thus, the decreased *Buchnera* levels in *Ac. gossypii* at G0 may reflect severe nutritional stress, contributing to reduced longevity and fecundity. The compensatory increase in *Buchnera* abundance in later generations of *Ac. gossypii* may represent a costly adaptive strategy. The host may need to invest additional resources to maintain the high abundance of *Buchnera*, which could potentially slow down other life activities such as development [65]. In contrast, the stable *Buchnera* community in *Ap. gossypii* suggests stronger basal tolerance or more efficient regulatory mechanisms, enabling it to cope with nitenpyram stress without perturbing its core symbiont. The mechanisms underlying these interspecific differences warrant further investigation.

*Sphingomonas*, widely distributed in the environment [68,69,70], has demonstrated degradation capabilities for phenyl-urea herbicide isoproturon [71], 5,5′-dehydrodivanillate [72], and pentachlorophenol [73]. Its marked response to nitenpyram stress in this study suggests a potential role in primary detoxification. Recent evidence confirms its presence in insect guts, including high abundance in *Spodoptera frugiperda* [74]. Here, nitenpyram significantly increased *Sphingomonas* abundance in both aphid species at G0. Given its documented role in mediating imidacloprid resistance in *Ap. gossypii* via enhanced metabolic degradation [75], the early rise in *Sphingomonas* implies its involvement in a coordinated host-symbiont defense response, potentially through direct insecticide metabolism or detoxification assistance. However, this putative detoxification role diverged across generations. In *Ac. gossypii*, *Sphingomonas* abundance declined sharply in G1 and normalized by G2, whereas *Ap. gossypii* maintained stable *Sphingomonas* populations throughout G1–G2. This divergence suggests species-specific adaptation strategies. *Ac. gossypii* may be unable to sustain the metabolic cost of maintaining high *Sphingomonas* levels, whereas *Ap. gossypii* likely stabilizes this beneficial microbial adjustment, possibly contributing to its higher population growth in offspring. Thus, the observed microbial shifts, particularly the *Sphingomonas* dynamics, represent a structured response. Its early increase provides strong indirect evidence of symbiotic involvement in insecticide detoxification. Future isolation and functional validation of these strains will clarify their contribution, further illuminating the key role of symbionts in aphid adaptation to insecticide stress.

In the present study, *Ac. gossypii* G0 generation *Acinetobacter* increased from 0.04% to 12.97%. It decreased from 29.06% and 32.72% to 0.15% and 0.22% in the G1 and G2 generations, respectively. In contrast, *Ap. gossypii* G1 generation *Acinetobacter* decreased compared to the control. Previous research has shown that *Acinetobacter* was isolated in the gut of pesticide-resistant *Helicoverpa armigera* [76]. Therefore, *Acinetobacter* may not be associated with resistance to *Ap. gossypii* and *Ac. gossypii* mediated by nitenpyram.

In summary, this study compares the effects of nitenpyram on the survival and life history traits of *Ap. gossypii* and the *Ac. gossypii*, while also examining its impact on their symbiotic bacteria. We found that nitenpyram exhibited greater toxicity toward *Ac. gossypii*. Following 48 h of exposure to nitenpyram, the longevity and fecundity of both aphid species were inhibited. Sublethal concentrations of the insecticide not only affected the parental generation but also influenced the offspring of both *Ap. gossypii* and *Ac. gossypii*. The *R*_0_, *r_m_*, and *λ* increased in *Ap. gossypii* but decreased in *Ac. gossypii*. Both aphid populations returned to stable levels by the third generation (G3). By analyzing the differences between toxicity and sublethal effects, we further investigated the impact of nitenpyram stress on the symbiotic bacteria of both aphid species. The results demonstrated that nitenpyram disrupted the stability of the symbiotic bacterial communities in both aphids. Two representative symbiotic bacteria, *Sphingomonas* and *Buchnera*, exhibited significant changes across three consecutive generations in both species. These findings enhance our understanding of *Ap. gossypii* and *Ac. gossypii* adaptation to insecticides and clarify the relationships between these two aphid species and their symbiotic bacteria under insecticide stress. This study provides a foundation for future integrated management strategies for cotton aphids.

It should be noted that a limitation of this study is the lack of functional validation of the potential detoxification mechanisms. We have therefore identified “elucidating the specific role in imidacloprid detoxification through in vitro symbiont culture and in vivo enzyme activity assays” as a key objective for future research.

## 5. Conclusions

This study systematically evaluated the effects of sublethal nitenpyram exposure on the survival, life history traits, and symbiotic bacterial communities of *Ap. gossypii* and *Ac. gossypii*. Nitenpyram exhibited greater toxicity to *Ac. gossypii* than to *Ap. gossypii*. Sublethal exposure for 48 h significantly reduced longevity and fecundity in the parental generation of both species, with transgenerational effects observed in their offspring. Population parameters (*R*_0_, *r_m_*, *λ*) increased in *Ap. gossypii* but decreased in *Ac. gossypii*, stabilizing by the third generation. Nitenpyram also disrupted the composition and stability of symbiotic bacteria, notably altering the relative abundances of *Sphingomonas* and *Buchnera* over three generations. These findings demonstrate species-specific and transgenerational shifts in aphid life history and microbial symbiosis under insecticide stress, providing insights for managing cotton aphid resistance.

## Figures and Tables

**Table 1:** Toxicity of nitenpyram to *Aphis gossypii* and *Acyrthosiphon gossypii* adults after 48 h exposure.

Insecticides	Aphid Species	LC_20_ (mg-L^−1^) (95%CL)	LC_50_ (mg-L^−1^) (95%CL)	Slope ± SE	*R* ^2^
Nitenpyram	*Ap. gossypii*	8.73 (6.04–11.49)	28.62 (22.50–37.64)	2.34 ± 0.26	0.98
	*Ac. gossypii*	2.49 (0.78–4.31)	10.12 (6.34–17.08)	1.41 ± 0.31	0.98

SE: Standard error; 95% CL: 95% confidence limits.

**Table 2:** Sublethal effects of nitenpyram on life table parameters of *Aphis gossypii* and *Acyrthosiphon gossypii* G1–G2.

Parameters	*Ap. gossypii*-G1			*Ac. gossypii*-G1			*Ap. gossypii*-G2			*Ac. gossypii*-G2		
	CK	Nitenpyram	*p*	CK	Nitenpyram	*p*	CK	Nitenpyram	*p*	CK	Nitenpyram	*p*
APOP	0.43 ± 0.09	0.13 ± 0.06	0.009	0.27 ± 0.08	0.00 ± 0.00	<0.001	0.43 ± 0.09	0.20 ± 0.07	0.046	0.13 ± 0.06	0.10 ± 0.05	0.690
TPOP	6.20 ± 0.18	5.93 ± 0.13	0.260	7.40 ± 0.14	8.03 ± 0.12	<0.001	6.30 ± 0.13	6.10 ± 0.11	0.231	7.17 ± 0.11	7.70 ± 0.14	0.004
Longevity	21.87 ± 0.47	20.93 ± 0.23	0.072	23.10 ± 0.28	22.03 ± 0.31	0.012	21.53 ± 0.42	21.87 ± 0.44	0.595	23.10 ± 0.22	21.97 ± 0.30	0.002
Fecundity	53.00 ± 2.03	58.40 ± 0.98	0.017	45.67 ± 1.10	39.83 ± 1.20	<0.001	49.37 ± 1.92	55.07 ± 1.80	0.032	44.80 ± 0.99	40.27 ± 1.03	0.001
*R*_0_	53.00 ± 2.03	58.40 ± 0.98	0.017	45.67 ± 1.10	39.83 ± 1.20	<0.001	49.37 ±1.92	55.07 ± 1.80	0.032	44.80 ± 0.99	40.27 ± 1.03	0.001
*r_m_*	0.36 ± 0.007	0.38 ± 0.005	0.022	0.31 ± 0.004	0.29 ± 0.004	<0.001	0.35 ± 0.005	0.37 ± 0.005	0.013	0.32 ± 0.004	0.30 ± 0.005	0.006
*λ*	1.43 ± 0.011	1.46 ± 0.008	0.021	1.36 ± 0.006	1.33 ± 0.005	<0.001	1.42 ± 0.007	1.44 ± 0.007	0.013	1.37 ± 0.006	1.35 ± 0.007	0.006
*T*	11.03 ± 0.26	10.69 ± 0.16	0.256	12.29 ± 0.19	12.88 ± 0.15	0.015	11.22 ± 0.17	10.98 ± 0.17	0.315	11.97 ± 0.16	12.34 ± 0.19	0.133

Note: Values in the table represent mean ± SE. The SE was calculated using the bootstrap technique with 100,000 resampling and differences among groups were compared by paired bootstrap test (*p* < 0.05). The SE was calculated using the bootstrap technique with 100,000 resampling and differences among groups were compared by paired bootstrap test (*p* < 0.05). (APOP) adult prereproductive period; (TPOP) total pre-reproductive period; (*R*_0_) net reproductive rate; (*r_m_*) intrinsic rate of increase; (*λ*) finite rate of increase; (*T*) mean generation time.

**Table 3:** Sublethal effects of nitenpyram on life table parameters of *Aphis gossypii* and *Acyrthosiphon gossypii* G3.

Parameters	*Ap. gossypii*-G3			*Ac. gossypii*-G3		
	CK	Nitenpyram	*p*	CK	Nitenpyram	*p*
APOP	0.33 ± 0.09	0.27 ± 0.08	0.671	0.07 ± 0.05	0.10 ± 0.05	0.810
TPOP	6.17 ± 0.13	6.13 ± 0.18	0.940	7.47 ± 0.12	7.20 ± 0.17	0.210
Longevity	22.03 ± 0.54	19.90 ± 0.44	0.003	22.13 ± 0.25	22.87 ± 0.25	0.044
Fecundity	54.70 ± 2.29	57.30 ± 1.70	0.361	43.17 ± 1.23	44.63 ± 0.90	0.337
*R*_0_	54.70 ± 2.29	57.30 ± 1.70	0.361	43.17 ± 1.23	44.63 ± 0.90	0.337
*r_m_*	0.37 ± 0.005	0.38 ± 0.009	0.286	0.31 ± 0.004	0.32 ± 0.005	0.070
*λ*	1.44 ± 0.007	1.46 ± 0.013	0.286	1.36 ± 0.006	1.38 ± 0.007	0.070
*T*	10.92 ± 0.17	10.72 ± 0.23	0.500	12.26 ± 0.17	11.90 ± 0.19	0.158

Note: Values in the table represent mean ± SE. The SE was calculated using the bootstrap technique with 100,000 resampling and differences among groups were compared by paired bootstrap test (*p* < 0.05). The SE was calculated using the bootstrap technique with 100,000 resampling and differences among groups were compared by paired bootstrap test (*p* < 0.05). (APOP) adult prereproductive period; (TPOP) total pre-reproductive period; (*R*_0_) net reproductive rate; (*r_m_*) intrinsic rate of increase; (*λ*) finite rate of increase; (*T*) mean generation time.

## Data Availability

The data presented in this study are available in the article.

## Supplementary Materials

The following supporting information can be downloaded at: https://www.mdpi.com/article/10.3390/biology14121684/s1, Table S1. Sequencing analysis of 16S rRNA of *Aphis gossypii* and *Acyrthosiphon gossypii;* Table S2. Bacterial community composition (%) of *Aphis gossypii* and *Acyrthosiphon gossypii* at different taxonomic levels in G0 generation (only showed proportion > 5%); Table S3. Bacterial community composition (%) of *Aphis gossypii* and *Acyrthosiphon gossypii* at different taxonomic levels in G1 generation (only showed proportion > 5%); Table S4. Bacterial community composition (%) of *Aphis gossypii* and *Acyrthosiphon gossypii* at different taxonomic levels in G2 generation (only showed proportion > 5%); Table S5. Relative abundances of the top 15 genus in G0 generation of *Aphis gossypii* and *Acyrthosiphon gossypii*; Table S6. Relative abundances of the top 15 genus in G1 generation of *Aphis gossypii* and *Acyrthosiphon gossypii*; Table S7. Relative abundances of the top 15 genus in G2 generation of *Aphis gossypii* and *Acyrthosiphon gossypii*.
