# Supplementary material for: Comparative Analysis of Symbiotic Bacterial Diversity and Sublethal Effects of Nitenpyram Against Two Different Cotton Aphids
Source: Biology (Basel). 2025 Nov 26;14(12):1684. doi: 10.3390/biology14121684 (PMC12730078; doi:10.3390/biology14121684)
Supplement: Supplementary file 1 [file biology-14-01684-s001.zip › biology-3993692-supplementary.pdf]

Supplementary Files

Table S1. Sequencing analysis of 16S rRNA of *Ap. gossypii* and *Ac. gossypii*

Aphid species	Samples	Raw_ reads	filtered	ASV _counts	chao1	Goods _coverage	shannon	Observed _species	simpson	ACE
Ap. gossypii	CK-G0	80961	78091	125	125.39	1.00	1.10	125.30	0.44	126.09
	N-G0	81090	77847	166	134.35	1.00	1.04	134.33	0.38	135.44
	CK-G1	80892	77885	134	115.68	1.00	1.12	115.60	0.45	116.55
	N-G1	80744	77320	128	166.02	1.00	1.25	165.93	0.48	166.26
	CK-G2	79822	76591	116	127.65	1.00	1.20	127.60	0.46	128.68
	N-G2	80964	78062	135	135.34	1.00	1.11	135.30	0.41	136.08
Ac. gossypii	CK-G0	79947	76925	229	228.96	1.00	1.22	228.60	0.27	228.06
	N-G0	79587	76499	315	314.75	1.00	3.86	314.63	0.72	316.08
	CK-G1	80480	77255	289	289.02	1.00	3.06	288.90	0.71	289.35
	N-G1	81553	78381	181	180.94	1.00	1.06	180.77	0.23	180.97
	CK-G2	79600	76584	247	247.34	1.00	2.37	247.30	0.64	247.43
	N-G2	79847	76721	220	220.54	1.00	1.10	220.23	0.22	220.24

Table S2. Bacterial community composition (%) of Ap. gossypii and Ac. gossypii at different taxonomic levels in G0 generation (only showed proportion > 5%)

Taxonomic categories	Taxa	*Ap. gossypii*			*Ac. gossypii*		
		P	Nitenpyram	CK	*P*	Nitenpyram	CK
Phylum	Proteobacteria	0.109	98.67	99.03	0.003	90.53	97.69
Class	Gammaproteobacteria	0.071	98.06	98.77	<0.001	78.63	96.60
Order	Enterobacterales	0.067	97.77	98.60	0.001	62.52	94.63
Family	Morganellaceae	0.082	97.61	98.46	0.001	62.16	94.31
Genus	*Buchnera*	0.168	62.84	69.11	<0.001	51.97	85.49
	Acinetobacter	-	-	-	0.003	12.97	0.04
	Arsenophonus	0.122	34.78	29.34	0.482	10.19	8.82

Table S3. Bacterial community composition (%) of *Ap. gossypii* and *Ac. gossypii* at different taxonomic levels in G1 generation (only showed proportion > 5%)

Taxonomic categories	Taxa	*Ap. gossypii*			*Ac. gossypii*		
		P	Nitenpyram	CK	*P*	Nitenpyram	CK
Phylum	Proteobacteria	0.586	99.03	98.96	0.011	98.10	95.53
Class	Gammaproteobacteria	0.452	98.70	98.57	0.001	96.79	88.64
Order	Enterobacterales	0.257	98.53	98.26	0.003	96.10	57.73
Family	Morganellaceae	0.174	98.39	98.02	<0.001	95.98	57.10
Genus	*Buchnera*	0.056	66.74	75.41	<0.001	87.67	47.42
	Acinetobacter	-	-	-	0.002	0.15	29.06
	Arsenophonus	0.072	31.65	22.61	0.131	8.31	9.68

Table S4. Bacterial community composition (%) of *Ap. gossypii* and *Ac. gossypii* at different taxonomic levels in G2 generation (only showed proportion > 5%)

Taxonomic categories	Taxa	*Ap. gossypii*			*Ac. gossypii*		
		P	Nitenpyram	CK	*P*	Nitenpyram	CK
Phylum	Proteobacteria	0.261	98.88	99.05	0.110	97.57	97.01
Class	Gammaproteobacteria	0.330	98.63	98.82	0.195	96.31	93.46
Order	Enterobacterales	0.698	98.20	98.44	0.008	95.63	52.72
Family	Morganellaceae	0.643	98.03	98.30	0.001	95.21	52.44
	Buchnera	0.245	73.05	66.68	0.009	88.14	46.97
Genus	*Acinetobacter*	-	-	-	0.001	0.22	32.72
	Arsenophonus	0.246	24.98	31.62	0.050	7.07	5.47

Table S5 Relative abundances of the top 15 genus in G0 generation of *Ap. gossypii* and *Ac. gossypii*

Taxonomy	*Ap. gossypii*			*Ac. gossypii*		
	P	Nitenpyram	CK	*P*	Nitenpyram	CK
Buchnera	0.168	62.84	69.11	<0.001	51.97	85.49
Arsenophonus	0.122	34.78	29.34	0.482	10.19	8.82
Muribaculaceae	0.326	0.20	0.25	0.951	0.14	0.14
Acinetobacter	0.629	0.06	0.10	0.003	12.97	0.04
Stenotrophomonas	-	-	-	0.380	0.03	1.52
Sphingomonas	0.010		0.06	0.002	6.18	0.17
[Ruminococcus]_gnavus_group	0.732		0.07	-	-	-
Enterobacter	0.895	0.15	0.05	0.769	0.10	0.07
Escherichia-Shigella	0.441	0.08	0.05	0.338	0.21	0.14
Lachnospiraceae_NK4A136_group	0.127	0.03	0.05		-	-
Bacteroides	0.020	0.06	0.03	0.026	0.48	0.13
SWB02	0.120	0.09	0.03	<0.001	0.63	0.16
Pseudomonas	-	-	-	0.016	0.10	0.02
Enterococcus	0.166	0.02	0.06	-	-	-
Comamonas	-	-	-	0.374	0.01	0.00
Paracoccus	-	-	-	0.001	0.13	0.00
TRA3-20	-	-	-	0.002	0.37	0.04
Blautia	0.644	0.02	0.03	-	-	-
Lactobacillus	0.125	0.05	0.02	-	-	-
Dongia	0.246	0.05	0.02	0.077	0.47	0.09
others	0.063	1.45	0.73	<0.001	16.02	3.17

Table S6 Relative abundances of the top 15 genus in G1 generation of *Ap. gossypii* and *Ac. gossypii*

Taxonomy	*Ap. gossypii*			*Ac. gossypii*		
	P	Nitenpyram	CK	*P*	Nitenpyram	CK
Buchnera	0.056	66.74	75.41	<0.001	87.67	47.42
Arsenophonus	0.072	31.65	22.61	0.131	8.31	9.68
Muribaculaceae	0.010	0.16	0.35	0.018	0.10	0.35
Acinetobacter	0.003	0.03	0.22	0.002	0.15	29.06
Stenotrophomonas	-	-	-	0.183	0.00	0.01
Sphingomonas	0.761	0.10	0.10	0.017	0.31	4.06
[Ruminococcus]_gnavus_group	0.722	0.08	0.07	-	-	-
Enterobacter	0.740	0.09	0.07	0.328	0.07	0.23
Escherichia-Shigella	0.264	0.03	0.07	0.002	0.03	0.29
Lachnospiraceae_NK4A136_group	0.109	0.06	0.03	-	-	-
Bacteroides	0.211	0.03	0.05	0.182	0.10	0.18
SWB02	0.757	0.03	0.03	0.718	0.14	0.18
Pseudomonas	-	-	-	0.111	0.01	0.14
Enterococcus	0.208	0.02	0.01	-	-	-
Comamonas	-	-	-	0.193	0.00	0.68
Paracoccus	-	-	-	0.076	0.00	0.42
TRA3-20	-	-	-	0.808	0.05	0.07
Blautia	0.005	0.04	0.01	-	-	-
Lactobacillus	0.541	0.03	0.02	-	-	-
Dongia	0.375	0.01	0.03	0.067	0.09	0.27
others	0.763	0.89	0.94	0.018	2.95	6.96

Table S7 Relative abundances of the top 15 genus in G2 generation of *Ap. gossypii* and *Ac. gossypii*

Taxonomy	*Ap. gossypii*			*Ac. gossypii*		
	P	Nitenpyram	CK	*P*	Nitenpyram	CK
Buchnera	0.245	73.05	66.68	0.009	88.14	46.97
Arsenophonus	0.246	24.98	31.62	0.050	7.07	5.47
Muribaculaceae	0.004	0.37	0.22	0.105	0.19	0.15
Acinetobacter	0.940	0.31	0.28	0.001	0.22	32.72
Stenotrophomonas	-	-	-	0.121	0.00	6.58
Sphingomonas	0.812	0.07	0.07	0.349	0.24	2.17
[Ruminococcus]_gnavus_group	0.057	0.04	0.09	-	-	-
Enterobacter	0.339	0.03	0.07	0.662	0.07	0.04
Escherichia-Shigella	0.003	0.06	0.03	0.612	0.21	0.18
Lachnospiraceae_NK4A136_group	0.331	0.06	0.04	-	-	-
Bacteroides	0.353	0.04	0.03	0.440	0.10	0.12
SWB02	0.277	0.03	0.02	0.639	0.13	0.10
Pseudomonas	-	-	-	0.285	0.01	0.80
Enterococcus	0.973	0.03	0.03	-	-	-
Comamonas	-	-	-	0.374	0.00	0.01
Paracoccus	-	-	-	0.287	0.02	0.07
TRA3-20	-	-	-	0.506	0.04	0.04
Blautia	0.199	0.03	0.04	-	-	-
Lactobacillus	0.324	0.02	0.01	-	-	-
Dongia	0.888	0.02	0.01	0.959	0.09	0.10
others	0.540	0.86	0.75	0.007	3.46	4.47
